# 

**Published:** 1889-09

**Authors:** 


					﻿Vol. IX.
SEPTEMBER,' 1889.
NO. 9.
THE
Homeopathic pH^iciAfl,
A Monthly Journal of Homoeopathic Materia Medica
and Clinical Medicine.
“ He is a freeman whom truth makes free, and all are slaves beside."
“Seek the Truth: come whence it may, cost what it will."
EDITED AND PUBLISHED BY
EDMUND J. LEE,
AND
Walter m. James, ml e>.
PHILADELPHIA :
No. 1133 SPRUCE STREET.
X.O2STT5O2T
ALFRED HEATH & CO., Sole AGENTS BOE Great Britain,
114 Ebury Street, Eaton Square.
BS'T’eariy Subscription, $2.50. invariably in advance. ■ Single numbers> 25 cts.
Single numbers containing the Repertory Supplement, 50 cts.
Entered at the Philadelphia Port-Office as Seoond- Claw Mail Matter.
				

